# Targeting IRES-Mediated p53 Synthesis for Cancer Diagnosis and Therapeutics

**DOI:** 10.3390/ijms18010093

**Published:** 2017-01-04

**Authors:** Bai Ji, Benjamin R. E. Harris, Yahui Liu, Yibin Deng, Sergio A. Gradilone, Margot P. Cleary, Jianhua Liu, Da-Qing Yang

**Affiliations:** 1 Department of Hepatobiliary and Pancreatic Surgery, the First Hospital of Jilin University, Changchun 130021, China; bji@hi.umn.edu (B.J.); jirulin@sina.com (Y.L.); 2 The Hormel Institute, University of Minnesota, 801 16th Avenue NE, Austin, MN 55912, USA; brharris@umn.edu (B.R.E.H.); yideng@hi.umn.edu (Y.D.); sgradilone@hi.umn.edu (S.A.G.); mpcleary@hi.umn.edu (M.P.C.); 3 The Masonic Cancer Center, University of Minnesota, Minneapolis, MN 55455, USA

**Keywords:** p53, internal ribosome entry site (IRES), DNA damage, IRES-trans acting factors (ITAFs)

## Abstract

While translational regulation of p53 by the internal ribosome entry site (IRES) at its 5′-untranslated region following DNA damage has been widely accepted, the detailed mechanism underlying the translational control of p53 by its IRES sequence is still poorly understood. In this review, we will focus on the latest progress in identifying novel regulatory proteins of the p53 IRES and in uncovering the functional connection between defective IRES-mediated p53 translation and tumorigenesis. We will also discuss how these findings may lead to a better understanding of the process of oncogenesis and open up new avenues for cancer diagnosis and therapeutics.

## 1. Introduction

The p53 tumor suppressor protein plays critical roles in preventing malignant transformation by inducing cell growth arrest or apoptosis [1]. Normally, p53 is inactive in the cell and its levels are low. In response to cellular stress such as DNA damage, p53 levels increase dramatically and it becomes activated through multiple post-translation modifications [2]. Although the induction of p53 is partly due to its stabilization through interaction with the ubiquitin ligase MDM2, it is now clear that newly synthesized p53 also contributes to its accumulation [3,4]. However, the induction of p53 following DNA damage is also accompanied by increased association between eIF-4E, a protein translation initiator that binds to 5′-cap of eukaryotic mRNA, and its inhibitory protein 4E-BP1. This indicates a decrease in cap-dependent translation following DNA damage. This observation has led to the discovery of an internal ribosomal entry site (IRES) at the 5′-untranslated region (UTR) of the p53 mRNA, which can recruit the 40S ribosomal subunit independent of the eIF-4E protein in response to DNA damage and other cellular stress [5]. The presence of the IRES sequence in the p53 mRNA was also found by a separate report [6]. Both studies have demonstrated that there are no cryptic splice sites or promoters in the IRES sequence (–131 to –1 nucleotides) of the p53 mRNA using multiple experimental approaches [5,6]. Since the discovery of the p53 IRES sequence within its 5′-UTR, there has been an abundance of inquiry into how to take advantage of it for cancer diagnostics and therapeutics. Recently, many regulatory proteins of the p53 IRES have been identified and their involvement in p53 induction and tumorigenesis has been studied [7,8]. This review article provides an update and a brief summary on the latest progress in this area of research.

## 2. A Switch from Cap-Dependent Translation to IRES-Mediated Translation of p53 mRNA Following DNA Damage and Discovery of the p53 IRES

The predominant form of eukaryotic protein synthesis is cap-dependent translation because eukaryotic mRNAs contain a 7-methylguanosine cap at their 5′-end. Before protein translation can begin, several steps are necessary for the formation of the translation initiation complex, eIF-4F. In the first step of the complex formation, eIF-4E is capable of recognizing and binding to the 5′-cap. A binding site on eIF-4E enables the scaffolding protein, eIF-4G, to attach and assemble the eIF-4F complex. Once this occurs, eIF-4F is able to bind the ternary complex tRNAMet-EIF2-GTP, which scans the mRNA 5′-UTR. The scanning process is assisted by eIF-4A, an RNA helicase bound to eIF-4G that unwinds inhibitory secondary structures in the mRNA. After reaching an AUG that is flanked by a favorable nucleotide context (Kozak sequence, [9]), the elongation phase of cap-dependent translation begins [10].

Conversely, the presence of 4E-BP1, a protein specifically binding to eIF-4E, inhibits cap-dependent translation by competitively binding to the same site through which eIF-4E associates with eIF-4G [10]. When insulin or other growth factors are available, 4E-BP1 is phosphorylated by multiple kinases [11,12]. Following phosphorylation, 4E-BP1 releases from eIF-4E, allowing eIF-4G to bind eIF-4E and initiate cap-dependent translation. However, the interaction between 4E-BP1 and eIF-4E occurs to block cap-dependent translation during conditions in which 4E-BP1 is hypophosphorylated. Under these circumstances, mRNAs that contain an IRES sequence in their 5′-UTR may undergo cap-independent translation. The IRES sequence is capable of recruiting the 40S ribosomal subunit without the participation of eIF-4E [3]. In this way, cells are able to express proteins necessary for critical functions such as growth arrest and apoptosis during cellular stress [13].

Several earlier reports found that p53 synthesis increases in response to DNA damage induced by ultraviolet (UV), ionizing radiation (IR), or various chemical carcinogens such as etoposide [5,14,15]. Etoposide mimics IR and induces DNA double-strand breaks within the cell. It was demonstrated that p53 biosynthesis increases rapidly in response to IR in mouse 3T3 cells, even after treating the cells with a transcription inhibitor [16]. Also, exposure to IR or etoposide was found to lead to an increase in the association of p53 mRNA with polysomes, which further indicates an increase in p53 mRNA translation [5,17]. However, we found a time-dependent increase in the association between 4E-BP1 and eIF-4E following etoposide-induced DNA damage, which suggests that increased p53 synthesis is not controlled by cap-dependent translation [5]. Using a bicistronic reporter, we discovered the presence of an IRES sequence in the p53 5′-UTR, which is able to drive cap-independent translation of p53 in response to DNA damage [5]. These findings thus not only uncover a shift from cap-dependent translation to IRES-mediated translation of p53 mRNA following DNA damage, but also provide novel insights into the mechanism underlying how p53 levels increase in response to DNA damage. 

The presence of an IRES sequence in the mRNA of ΔN-p53, an isoform of p53 (also known as p53/p47, Δ40p53, and p47), and the mRNA of a p53 homologue, p73, has also been discovered [6,18,19]. The IRES of ΔN-p53 mRNA is a 117-nucleotide sequence downstream of the 1st AUG of the p53 mRNA that contains no internal promoters or cryptic splice sites [6]. While it is still unclear as to how ΔN-p53 regulates cell growth and cell death under different stressful conditions, accumulating evidence suggests that ΔN-p53 can induce apoptosis, either alone or in combination with p53, by activating the transcription of several apoptotic proteins not controlled by p53 [20]. It was recently found that mouse p53 mRNA also contains an IRES sequence [21], which is consistent with a previous finding that mouse p53 synthesis is up-regulated by DNA damage [16]. 

More recently, several lines of new evidence have further confirmed the role of the p53 IRES in stimulating p53 synthesis in response to other types of cellular stress [22,23,24,25,26,27]. For example, it is known that mouse embryo fibroblasts transduced with retroviruses of activated Ras oncogene initially undergo increased proliferation [24]. This increased proliferation was accompanied by an increase in cap-dependent translation. Oncogene-induced senescence (OIS) is a rapid cellular response that permanently shuts down further proliferation to prevent malignant transformation. It was shown that cap-dependent translation switches to IRES-mediated cap-independent translation of p53 during OIS [24]. Specifically, the p53 IRES exhibits enhanced activity following OIS, which leads to an increase in p53 accumulation as cap-dependent translation stops [24]. More recently, it was shown that IRES activity of p53 increases in response to glucose deprivation, which links p53 IRES activity with metabolic stress [27] and provides further evidence that the p53 IRES plays a key role in regulation of p53 synthesis following various types of cellular stress. 

## 3. Identification of p53 ITAFs That Regulate IRES-Mediated Translation of p53 mRNA 

The IRES structure, in addition to binding canonical initiation factors such as eIF-4G, may also serve as an anchoring point for auxiliary protein initiation factors known as IRES trans-acting factors, or ITAFs. These ITAFs modulate activity of the IRES, either positively or negatively [28,29]. The positive ITAFs may act as RNA chaperones, remodeling and opening up the complex secondary structure of the IRES to allow binding of the ribosomal subunits to the mRNA, thereby facilitating translation initiation [29]. The negative ITAFs may preserve or further stabilize the secondary structure of the IRES and inhibit mRNA translation. In response to DNA damage or other cellular stress, negative ITAFs will dissociate from the p53 mRNA to allow positive ITAFs and ribosomal binding to the p53 IRES [29]. 

In one of the initial attempts searching for ITAFs of p53 IRES, Grover et al. found that the polypyrimidine-tract-binding protein (PTB) specifically binds to the IRES sequences of p53 and ΔN-p53 mRNAs as a positive ITAF, with a preference of ΔN-p53 mRNA [22]. Following DNA damage, PTB translocates from nucleus to the cytoplasm, and increased cytoplasmic abundance of PTB contributes to elevated p53 and ΔN-p53 IRES activity, leading to increased synthesis of p53 and ΔN-p53 [22]. Later, the same research group identified two new ITAFs, Annexin A2 and PTB associated Splicing Factor (PSF/SFPQ), that positively regulate the p53 IRES activity. Using an RNA affinity assay, they found that Annexin A2 and PSF proteins specifically bind to the p53 IRES. Partial knockdown of Annexin A2 and PSF results in decreased p53 IRES activity and reduced levels of p53. More interestingly, these two proteins have overlapping binding site of the p53 IRES as knockdown of one protein inhibits the other’s ability to interact with p53 IRES [25]. As PSF also associates with PTB, the interplay between PTB, PSF, and Annexin A2 appears to play an important role in the function of both p53 and ΔN-p53 IRESs. Recently, eIF-4G homolog death associated protein 5 (DAP5), an auxiliary translation initiation factor known to positively regulate multiple IRES containing mRNAs, was found to stimulate IRES-mediated translation of p53 and ΔN-p53 mRNAs, with ΔN-p53 mRNA as its main target [26]. 

An earlier study identified a number of proteins bound to the p53 5′-UTR in vitro [30]. Many of them are known to be involved in multiple cellular events, including protein translation and ribosomal biogenesis. Among these proteins, we discovered two novel ITAFs of p53, translational control protein 80 (TCP80) and RNA helicase A (RHA) , which positively regulate p53 IRES activity and p53 synthesis [7,8]. Both TCP80 and RHA are RNA-binding proteins and have documented roles in protein translation [31,32,33]. We found that TCP80 binds to the p53 IRES in vivo, possibly through one or both of its double-stranded RNA binding motifs. We also observed increased binding of TCP80 to the p53 IRES following DNA damage [8]. Moreover, overexpression of TCP80 leads to increased p53 IRES activity in response to DNA damage [8]. Similarly, we also found that overexpression of RHA increases p53 IRES activity and p53 synthesis, and knockdown of this protein leads to reduced p53 IRES activity and p53 levels in the cell [7]. 

**Figure 1:**
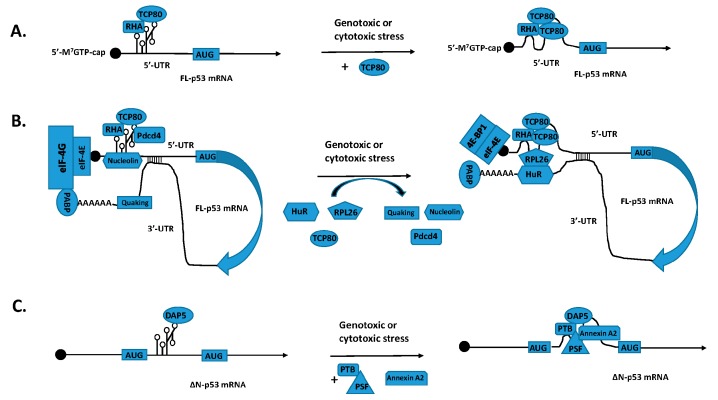
Regulation of full-length p53 mRNA (FL-p53 mRNA) and ΔN-p53 mRNA translation by their IRES sequences following geno- and cytotoxic stress. (**A**) A schematic representation showing regulation of p53 IRES activity by TCP80 and RHA. During the basal conditions, the secondary structure of the p53 IRES is stabilized and has limited translational activity, partly due to the inadequate interaction between TCP80/RHA and the p53 IRES. Following DNA damage and other cellular stress, increased binding of TCP80 to the p53 IRES and enhanced interaction between TCP80/RHA and the p53 IRES facilitates the unwinding of the secondary structure of the p53 IRES, allowing enhanced translation of the FL-p53 mRNA; (**B**) A putative, comprehensive model of regulation of p53 IRES activity by both its ITAFs and 3′-UTR binding proteins. During the basal conditions, the secondary structure of the p53 IRES is stabilized and has limited translational activity, due to its binding of several putative negative p53 ITAFs (nucleolin, Pdcd4, and FBL, etc.), inadequate interaction between TCP80/RHA, and the binding of p53 3′-UTR by its negative regulatory proteins, such as Quaking, despite the presence of the cap-dependent translational machinery (eIF-4E, eIF-4G, etc.) and PABP (poly A binding protein) that interacts with eIF-4G. Following DNA damage and other cellular stress, several positive p53 ITAFs (RPL26, TCP80, hnRNP Q, etc.) have displaced negative ITAFs bound to p53 IRES. These positive ITAFs bind to the p53 IRES and facilitate the unwinding of its secondary structure, resulting in enhanced translation of the FL-p53 mRNA. The unwinding of the p53 IRES secondary structure is also assisted by interaction between positive p53 ITAFs and other positive regulatory proteins, such as HuR, that are bound to the p53 3′-UTR after displacing Quaking in response to geno- or cytotoxic stress. The circulation of 5′- and 3′-UTR of the FL-p53 mRNA is stabilized by this interaction, despite disruption of the binding between eIF-4E and eIF-4G by increased amounts of 4E-BP1 following DNA damage and other cellular stress; (**C**) A diagram illustrating regulation of ΔN-p53 IRES activity by its positive ITAFs. During the basal conditions, the secondary structure of the ΔN-p53 IRES is stabilized and has limited translational activity. Following DNA damage and other cellular stress, PTB translocates from nuclear to the cytoplasm, binding of PTB/PSF and Annexin A2 to the IRES of ΔN-p53 mRNA facilitates the unwinding of the secondary structure of the ΔN-p53 IRES, resulting in increased translation of the ΔN-p53 mRNA.

While TCP80 is an RNA-binding protein and contains multiple RNA binding domains [31], RHA is an RNA helicase. Since the region containing the p53 IRES is known to have a strong secondary structure [30], it is not surprising that both TCP80 and RHA exert a positive effect on the p53 IRES by aiding in the unwinding of its secondary structure. Furthermore, TCP80 and RHA proteins were found to bind to each other in vitro [18]. We have further confirmed that these two proteins associate with each other in vivo [8]. More importantly, we also observed increased binding of TCP80 to RHA following DNA damage, and overexpression of TCP80, along with RHA, leads to increased expression of p53 than TCP80 or RHA alone [8]. These results suggest that the interaction between TCP80 and RHA is important for the stimulation of p53 IRES activity and p53 induction following DNA damage. The cooperative effect of TCP80 and RHA could be that RHA utilizes its ability to remodel RNA-RNA or RNA-protein interactions to facilitate increased binding of TCP80 to the p53 IRES following DNA damage, therefore leading to increased p53 IRES activity. Additionally, once more TCP80 is bound to p53 IRES, this may further facilitate the interaction between RHA and p53 IRES and help unwind the secondary structure of the p53 IRES [8]. Diagrams that summarize regulation of translation of the full-length p53 mRNA (FL-p53 mRNA) and the ΔN-p53 mRNAs are shown in Figure 1A,C, respectively.

In addition, among the over 20 proteins bound to p53 5′-UTR identified in Takagi et al.’s report, two other proteins, ribosomal protein L26 (RPL26) and nucleolin, were found to either positively or negatively affect p53 induction, respectively [30]. RPL26 and nucleolin bind to the 5′-UTR of p53 mRNA and control p53 induction and synthesis after DNA damage. RPL26 binds to the 5′-UTR after DNA damage, and its overexpression enhances association of p53 mRNA with polysomes and increases the rate of p53 mRNA translation. As a result, overexpression of RPL26 induces G1 cell-cycle arrest and augments irradiation-induced apoptosis [30]. In contrast, overexpression of nucleolin suppresses p53 synthesis and DNA damage-mediated p53 induction, whereas reducing endogenous nucleolin levels within the cell was able to restore IR-induced increase of p53. Interestingly, RPL26 and nucleolin were also shown to compete for the same binding site on the p53 IRES [30]. 

Recently, multiple other putative p53 ITAFs were found to bind to p53 5′-UTR and affect levels of p53. Some proteins, such as hnRNP Q [21], positively regulate the p53 induction, whereas others, including Pdcd4 [34] and FBL [35], negatively regulate levels of p53. While it remains to be determined whether these proteins, as are RPL26 and nucleolin, are bona fide p53 ITAFs, it will be important to investigate if they interact with other known p53 ITAFs or the 3′-UTR binding proteins (see below) to regulate IRES-mediated translation of p53 mRNA. 

## 4. Regulation of p53 mRNA Translation by its 3′-UTR through the Interaction between its 5′- and 3′-UTR binding proteins

In addition to the discovery of many proteins that regulate p53 translation through binding to the p53 IRES or its 5′-UTR, it was reported that the 3′-UTR of p53 mRNA also plays an important role in regulating p53 translation in response to DNA damage [36]. Mathematical modeling predicts formation of circularization of p53 mRNA through binding of its 5′-UTR and 3′-UTR [37]. Indeed, an interaction between the 5′-UTR (~130 nt, harboring the p53 IRES sequence) and 3′-UTR of the p53 mRNA (~1170 nt) has been uncovered [37]. It was found that addition of the p53 3′-UTR to a luciferase reporter containing p53 5′-UTR increases the function of RPL26, further enhancing the amount of firefly luciferase produced compared to the vector containing 5′-UTR alone [37]. Further evidence shows that disrupting this interaction or circulation between 5′- and 3′-UTR of p53 mRNA via oligonucleotides complementary to either UTR abrogates RPL26’s ability to enhance p53 translation [37].

Furthermore, it was shown that proteins bound to the p53 3′-UTR also modulate p53 synthesis following DNA damage [15]. Several proteins that can bind to the 3′-UTR of p53 mRNA and modulate its translation were identified [15,36,38]. An RNA-binding protein, HuR, was shown to mediate the UVC-induced increase in p53 translation by binding to the 3′-UTR of p53 mRNA following UVC irradiation [15]. Proteins including Quaking, the human homolog of GDL-1 [38], and an unknown 40 kDa protein [36] were found to bind the 3′-UTR of p53 mRNA and inhibit p53 synthesis. It is thought that proteins such as Quaking bind to the 3′-UTR and inhibit p53 mRNA translation under normal growth conditions, and DNA damage could let these proteins dissociate from the 3′-UTR and allow binding of HuR [15].

The 3′-UTRs of certain mRNAs have been shown to stimulate viral IRES-mediated translation [39]. Thus, the model of circular mRNA can be used to explain the reasons why proteins bound to the 3′-UTR affect the translation of mRNAs containing an IRES sequence. It is conceivable that proteins bound to the 3′-UTR may interact with ITAFs that bind to the 5′-UTR to either destabilize/open up the IRES secondary structure or further stabilize the structure, thereby facilitating or inhibiting IRES-mediated p53 mRNA translation, respectively. A putative, comprehensive model of regulation of p53 IRES activity by both its ITAFs and 3′-UTR binding proteins is illustrated in Figure 1B.

## 5. Altered Expression of p53 ITAFs in Cancer Cells with Defective Induction of p53 and Its Implication in Tumorigenesis and Cancer Diagnosis 

p53 is the most commonly mutated gene (>50%) in cancer. Thus, the majority of p53-related studies are focused on mutated p53 which has already lost its transcriptional activity. However, a number of malignancies, such as breast, prostate, and liver cancer, have a much lower rate of p53 mutation (18%–20%) [40,41] (also see http://p53.free.fr). While it is unclear why 80% or more of these tumors develop malignancy even in the presence of wild-type p53, very limited studies have been done to date to investigate the role of defective p53 mRNA translation in tumorigenesis process.

An earlier report shows that the lack of p53 synthesis is responsible for the abrogation of p53 accumulation following DNA damage and the development of malignant peripheral nerve sheath tumors (MPNST) in zebrafish [42]. In cells from heterozygote zebrafish with ribosomal protein mutations, p53 protein levels do not increase as a result of gamma-irradiation-induced DNA damage, regardless of detectable wild-type p53 mRNA within the cells. It was shown that this lack of p53 synthesis is responsible for the abrogated p53 accumulation following the DNA damage and leads to the development of MPNST [42].

A recent study observed defective IRES-mediated p53 synthesis in X-linked dyskeratosis congenita (X-DC), a tumor susceptible syndrome, in response to DNA damage and OIS [24]. X-DC is caused by mutations in the DKC1 gene and is characterized by bone marrow failure, ribosomal dysfunction, and increased susceptibility to cancer. The DKC1 gene encodes dyskerin, a pseudouridine synthase that modifies ribosome RNA [43]. It was found that mutation of the DKC1 gene leads to aberrant cellular IRES-mediated translation [44]. In particular, IRES-mediated p53 mRNA translation is defective following DNA damage in these cell lines [24]. It is also important to note that the switch from cap-dependent to IRES-mediated cap-independent translation also does not occur in cells derived from patients with X-DC.

Furthermore, another study revealed a direct link between IRES-mediated p53 synthesis and the aggressiveness of breast cancer. It was found that knockdown of dyskerin is associated with low levels of IRES-mediated p53 synthesis in human breast cancer cells, which results in p53 inactivation [23]. More importantly, in a number of human primary breast cancers expressing wild-type p53, low levels of dyskerin expression were associated with reduced expression of p53 downstream target genes [23]. 

As stated above, multiple new ITAFs or putative ITAFs of the p53 IRES have been identified and have been shown to affect the synthesis of p53 and its isoform ∆N-p53 under different stressful conditions [22,25,26,27]. Interestingly, among nearly twenty identified p53 5′-UTR binding proteins, many of them, including TCP80, RHA, RPL26 and nucleolin, are involved in either protein translation or ribosomal biogenesis [30]. However, until recently, it is unclear how dysfunction and/or altered expression/localization of these ITAFs link to defective p53 induction and cancer development. 

In our recent study, we found two breast cancer cell lines, MDA-MB-175 and ZR-75-1, that express wild-type p53 protein but exhibit diminished p53 induction and synthesis following DNA damage [7]. Our results also show that p53 IRES activity cannot be further enhanced by DNA damage in these two cell lines. Since earlier studies demonstrated that IRES/ITAF interactions could be important regulators of tumorigenesis, caused by either mutations in the cellular IRES sequences or altered expression of cellular ITAFs [29,45], we first sequenced the p53 mRNA in these two cell lines, which revealed that they retain wild-type p53 5′-UTR [7]. 

However, we found that expression of TCP80 and RHA (both are positive ITAFs of p53) is extremely low in ZR75-1 and MDA-MB-175 cells as compared to MCF-7 cells [7]. In contrast to ZR75-1 and MDA-MB-175, MCF-7 cells exhibit normal p53 response, including p53 induction and synthesis, following DNA damage [5]. Interestingly, while neither TCP80 nor RHA alone strongly stimulates the p53 IRES activity in these cell lines, expression of both TCP80 and RHA is required to significantly increase the p53 IRES activity [7]. Thus, these results suggest that defective p53 induction and synthesis are caused by reduced expression of TCP80 and RHA in ZR75-1 and MDA-MB-175 cells. Moreover, we found that knockdown of TCP80 in MCF-7 cells (MCF-7/shTCP80) results in decreased expression of RHA. In fact, with reduced expression of TCP80 and RHA, MCF-7/shTCP80 cells exhibit decreased induction of p53 and its downstream target p21^Cip1^ following DNA damage, which results in diminished ability to induce cellular senescence [7]. Furthermore, MCF-7/shTCP80 cells display defective induction of the p53 upregulated modulator of apoptosis (PUMA) [46,47], a pro-apoptotic protein whose expression and activity are regulated by p53 [8]. As both cellular senescence and apoptosis are critical in preventing the development of malignancies [48], these results provide a sound explanation in terms of why both ZR75-1 and MDA-MB-175 develop into cancer cells despite harboring wild-type p53. 

It is known that RHA maps to chromosome band 1q25, the site of a major prostate cancer susceptibility locus [49]. RHA also up-regulates several other tumor suppressors, including Werner Syndrome Helicase (WRN), that are involved in the DNA repair process [50,51]. Thus, it is likely that alterations of this locus may result in abrogated RHA function and prevent induction of p53 and other tumor suppressors, thereby increasing the risk of tumorigenesis. In addition, TCP80 expression is found to be reduced in malignant brain tumors of glial origin and the subcellular localization of TCP80 is also altered in these tumors [52]. These results provide further support that abnormal expression or subcellular localization of TCP80 or RHA is linked to malignant transformation. Thus, a decrease in the expression of positive p53 ITAFs or the overexpression of negative p53 ITAFs may cause reduced p53 induction and malignant transformation of cells harboring wild-type p53. Since altered expression of p53 ITAFs may occur well before the malignant transformation, the abnormal expression of negative and positive p53 ITAFs could be used as biomarkers for early diagnosis of cancer. 

## 6. Targeting IRES-Mediated p53 Synthesis for Cancer Therapeutics

As mentioned above, despite being most commonly mutated gene in cancer, wild-type p53 is still retained in the majority (~80%) of certain types of cancers, including breast cancer, prostate cancer and liver cancer. In addition, unlike the majority of adult cancers, pediatric tumors, including neuroblastoma, pediatric sarcomas and gliomas, still harbor wild-type p53 [53]. Because of this, searching for small molecules that can reactivate p53 in cancer cells that retain wild-type p53 has been an intensive area of research [54].

For cancer cells with wild-type p53, one of the important mechanisms of p53 inactivation is the amplification of the MDM2 gene. MDM2 protein is a main regulator of p53 stabilization. The p53 protein is maintained at low levels during normal conditions because MDM2 functions as a p53-selective E3-ubiquitin ligase that promotes p53 polyubiquitination and targets it for proteasomal degradation [2]. Upon DNA damage, p53 is phosphorylated at Ser15, which results in the dissociation of MDM2 from p53, leading to increased levels of p53 [54].

To date, the best-characterized MDM2/p53 antagonist is the cis-imidazoline derivative, termed Nutlin-3, and its analogs [55]. Restoration of p53 activity by Nutlin-3 or its analogs has been shown to induce potent anti-proliferation effects in various types of childhood cancer cell lines that overexpress MDM2 [53]. Preclinical studies using animal cancer models also showed that Nutlin-3 is effective against a variety of cancer cells with wild-type p53, including neuroblastoma, retinoblastoma, osteosarcoma and leukemia [56]. 

However, it was known that continuous exposure to Nutlin-3 can lead to the acquisition of somatic mutations in p53 and select for p53-mutated cells both in neuroblastoma and in other solid tumors such as osteosarcoma [57]. A recent study using improved methods of detecting MDM2 amplification suggests low frequencies (~10%) of MDM2 gene amplification in the majority of various types of cancers, including those in childhood [58]. This is probably why clinical trials testing Nutlin-3 and its analogs have only seen limited success [59]. Novel approaches for reactivating p53 in cancer cells expressing wild-type p53 are urgently needed. 

As stated in the above section, we discovered a novel mechanism of p53 inactivation, caused by dysregulation of p53 mRNA translation in cancer cells [7]. We found defective p53 induction following DNA damage in both ZR75-1 and MDA-MB-175 breast cancer cell lines. However, this deregulation is not related to abnormal MDM2 function as our results show that p53 is stabilized in ZR-75-1 cells and the half-life of p53 is similar in both MCF-7 and MDA-MB-175 cells [7]. Our results further indicate that defective p53 induction is caused by reduced expression of the positive p53 ITAFs TCP80 and RHA, which leads to diminished IRES-mediated p53 synthesis [7]. 

Therefore, p53 ITAFs, such as TCP80 and RHA, could become promising therapeutic targets for cancer treatment aimed at increasing levels of p53. For example, chemical compounds could be developed or identified by screening small molecules that specifically increase expression of positive p53 ITAFs or inhibit expression of negative p53 ITAFs, thereby stimulating p53 translation in cancer cells. Addition of these compounds in cancer cells may specifically induce p53, leading to cell growth arrest or apoptosis. 

In a previous study, we also discovered a switch from cap-dependent translation to IRES-mediated translation of p53 mRNA following DNA damage [5], which is accompanied by increased binding between 4E-BP1 and eIF-4E. A recent study made a similar finding in which a transition from cap-dependent translation to IRES-mediated synthesis of the p53 protein occurs in response to OIS [24].

It is known that eIF-4E levels and cap-dependent translation are frequently elevated in malignancies of the prostate, lung, breast, as well as many other organs [60]. As a consequence, overexpression and hyperactivation of eIF-4E cause malignant transformation and metastasis in various cancers [60]. Notably, in several types of cancers such as head and neck and breast cancers, levels of eIF-4E correlate with disease progression and poor prognosis [60]. These observations support the notion that cancers need to sustain activation of eIF-4E to drive translation of a subset of mRNAs required for the malignant phenotype. Thus, targeting elevated eIF-4E function in cancer cells has become an attractive therapeutics strategy for malignancies. 

Several small molecule cap-dependent inhibitors have recently been developed. One such chemical is 4Ei-1 (N-7 Benzyl Guanosine Monophosphate Tryptamine Phosphoramidate Pronucleotide), a cell-permeable antagonist of eIF-4E [61]. 4Ei-1 is a pro-drug that is converted to 7Bn-GMP by the enzyme histidine triad nucleotide binding protein-1 (HINT1) [62]. 7Bn-GMP is an analog to the M^7^GTP cap structure and is able to compete with the 5′-mRNA cap for binding to eIF-4E, preventing the recruitment of the 5′-mRNA cap structure to the eIF-4F complex [63]. It was reported that 4Ei-1 can reduce proliferation and repress colony formation of cancer cells by inhibiting eIF-4E function [63]. 

4EGI-1 is a selective inhibitor that directly binds to eIF-4E and disrupts the eIF-4E/eIF-4G interaction, thereby inhibiting cap-dependent translation [64]. 4EGI-1 was shown to have strong anti-proliferation and anti-metastasis effects in several types of cancer cells but appears to spare normal cells [64,65,66]. A recent report has demonstrated that 4EGI-1 binds to a site of eIF-4E that is distant from its eIF-4G binding site, and it disrupts eIF-4E and eIF-4G binding allosterically without affecting the binding between 4E-BP1 and eIF-4E [67]. 

While 4Ei-1 and 4EGI-1 are strong inhibitors of cap-dependent translation, neither inhibitor suppresses cap-independent protein translation. In fact, both 4Ei-1 and 4EGI-1 stimulate cellular IRES activity [61,64], which agrees with our finding that inhibition of cap-dependent translation actually leads to a switch to cap-independent translation of p53. Multiple lines of recent evidence have shown that inhibiting eIF-4E function in cancer cells lead to cell apoptosis [65,68]. However, the underlying mechanism for induction of apoptosis through inhibition of cap-dependent translation is unclear. Therefore, it will be interesting to explore whether treatment of cancer cells with inhibitors of cap-dependent translation causes a similar transition from cap-dependent translation to IRES-mediated translation of p53, leading to apoptosis. 

DNA damaging agents, such as etoposide, are commonly used in cancer chemotherapy. However, these agents also cause serious collateral damage to benign cells, which leads to increased risks of development of future cancer in treated individuals [54]. Since the majority of the traditional chemotherapeutic reagents induce DNA damage by triggering a p53 response [54], cap-dependent inhibitors or chemical compounds that specifically regulate expression of p53 ITAFs may replace some of these DNA damaging agents in cancer treatment, which may reduce toxic side effect and drug resistance causing by DNA damaging drugs in chemotherapy. It is also conceivable that these inhibitors or chemical compounds may be combined with MDM2-p53 interaction inhibitors and/or DNA damaging agents to increase the efficacy of p53-basad chemotherapy since they employ distinct mechanisms in inducing p53 in cancer cells. 

## 7. Conclusions

To date, the majority of research on p53 has been aimed at characterizing genetic mutations or posttranslational modifications that alter the p53 protein and lead to the loss of its transcriptional activity or its induction in cancer cells [1]. Despite the importance of p53 mRNA translation in initiating the events leading to cell growth arrest and apoptosis, the mechanisms underlying translational regulation of p53 and the role of defective p53 synthesis in tumorigenesis are still significantly understudied [3]. While the detailed mechanisms behind IRES-mediated p53 synthesis and the link between defective p53 mRNA translation and tumorigenesis still remain to be further elucidated, the discovery of the regulatory proteins of p53 IRES and their defective expression in cancer cells has opened the door for exploring novel biomarkers for cancer diagnosis and new therapeutic avenues for reactivating p53 in cancer cells.

## References

[1] Vogelstein B., Lane D., Levine A.J. (2000). Surfing the p53 network. Nature.

[2] Giaccia A.J., Kastan M.B. (1998). The complexity of p53 modulation: Emerging patterns from divergent signals. Genes Dev..

[3] Halaby M.J., Yang D.Q. (2007). p53 translational control: A new facet of p53 regulation and its implication for tumorigenesis and cancer therapeutics. Gene.

[4] Grover R., Candeias M.M., Fahraeus R., Das S. (2009). p53 and little brother p53/47: Linking IRES activities with protein functions. Oncogene.

[5] Yang D.Q., Halaby M.J., Zhang Y. (2006). The identification of an internal ribosomal entry site in the 5′-untranslated region of p53 mRNA provides a novel mechanism for the regulation of its translation following DNA damage. Oncogene.

[6] Ray P.S., Grover R., Das S. (2006). Two internal ribosome entry sites mediate the translation of p53 isoforms. EMBO Rep..

[7] Halaby M.J., Harris B.R., Miskimins W.K., Cleary M.P., Yang D.Q. (2015). Deregulation of IRES-mediated p53 translation in cancer cells with defective p53 response to DNA damage. Mol. Cell Biol..

[8] Halaby M.J., Li Y., Harris B.R., Jiang S., Miskimins W.K., Cleary M.P., Yang D.Q. (2015). Translational Control Protein 80 Stimulates IRES-Mediated Translation of p53 mRNA in Response to DNA Damage. BioMed Res. Int..

[9] Kozak M. (1987). An analysis of 5′-noncoding sequences from 699 vertebrate messenger RNAs. Nucleic Acids Res..

[10] Gingras A.C., Raught B., Sonenberg N. (1999). eIF4 initiation factors: Effectors of mRNA recruitment to ribosomes and regulators of translation. Annu. Rev. Biochem..

[11] Yang D., Brunn G.J., Lawrence J.C. (1999). Mutational analysis of sites in the translational regulator, PHAS-I, that are selectively phosphorylated by mTOR. FEBS Lett..

[12] Yang D.Q., Kastan M.B. (2000). Participation of ATM in insulin signalling through phosphorylation of eIF-4E-binding protein 1. Nat. Cell Biol..

[13] Holcik M., Sonenberg N. (2005). Translational control in stress and apoptosis. Nat. Rev. Mol. Cell Biol..

[14] Kastan M.B., Onyekwere O., Sidransky D., Vogelstein B., Craig R.W. (1991). Participation of p53 protein in the cellular response to DNA damage. Cancer Res..

[15] Mazan-Mamczarz K., Galban S., Lopez de Silanes I., Martindale J.L., Atasoy U., Keene J.D., Gorospe M. (2003). RNA-binding protein HuR enhances p53 translation in response to ultraviolet light irradiation. Proc. Natl. Acad. Sci. USA.

[16] Mosner J., Mummenbrauer T., Bauer C., Sczakiel G., Grosse F., Deppert W. (1995). Negative feedback regulation of wild-type p53 biosynthesis. Embo J..

[17] Fu L., Benchimol S. (1997). Participation of the human p53 3′UTR in translational repression and activation following gamma-irradiation. Embo J..

[18] Candeias M.M., Powell D.J., Roubalova E., Apcher S., Bourougaa K., Vojtesek B., Bruzzoni-Giovanelli H., Fahraeus R. (2006). Expression of p53 and p53/47 are controlled by alternative mechanisms of messenger RNA translation initiation. Oncogene.

[19] Sayan A.E., Roperch J.P., Sayan B.S., Rossi M., Pinkoski M.J., Knight R.A., Willis A.E., Melino G. (2007). Generation of DeltaTAp73 proteins by translation from a putative internal ribosome entry site. Ann. N. Y. Acad. Sci..

[20] Sharathchandra A., Katoch A., Das S. (2013). IRES mediated translational regulation of p53 isoforms. Wiley Interdiscip. Rev. RNA.

[21] Kim D.Y., Kim W., Lee K.H., Kim S.H., Lee H.R., Kim H.J., Jung Y., Choi J.H., Kim K.T. (2013). hnRNP Q regulates translation of p53 in normal and stress conditions. Cell Death Differ..

[22] Grover R., Ray P.S., Das S. (2008). Polypyrimidine tract binding protein regulates IRES-mediated translation of p53 isoforms. Cell Cycle.

[23] Montanaro L., Calienni M., Bertoni S., Rocchi L., Sansone P., Storci G., Santini D., Ceccarelli C., Taffurelli M., Carnicelli D. (2010). Novel dyskerin-mediated mechanism of p53 inactivation through defective mRNA translation. Cancer Res..

[24] Bellodi C., Kopmar N., Ruggero D. (2010). Deregulation of oncogene-induced senescence and p53 translational control in X-linked dyskeratosis congenita. Embo J..

[25] Sharathchandra A., Lal R., Khan D., Das S. (2012). Annexin A2 and PSF proteins interact with p53 IRES and regulate translation of p53 mRNA. RNA Biol..

[26] Weingarten-Gabbay S., Khan D., Liberman N., Yoffe Y., Bialik S., Das S., Oren M., Kimchi A. (2013). The translation initiation factor DAP5 promotes IRES-driven translation of p53 mRNA. Oncogene.

[27] Khan D., Katoch A., Das A., Sharathchandra A., Lal R., Roy P., Das S. (2015). Reversible induction of translational isoforms of p53 in glucose deprivation. Cell Death Differ..

[28] Hellen C.U., Sarnow P. (2001). Internal ribosome entry sites in eukaryotic mRNA molecules. Genes Dev..

[29] Stoneley M., Willis A.E. (2004). Cellular internal ribosome entry segments: Structures, trans-acting factors and regulation of gene expression. Oncogene.

[30] Takagi M., Absalon M.J., McLure K.G., Kastan M.B. (2005). Regulation of p53 translation and induction after DNA damage by ribosomal protein L26 and nucleolin. Cell.

[31] Fierro-Monti I., Mathews M.B. (2000). Proteins binding to duplexed RNA: One motif, multiple functions. Trends Biochem. Sci..

[32] Xu Y.H., Grabowski G.A. (1999). Molecular cloning and characterization of a translational inhibitory protein that binds to coding sequences of the human acid beta-glucosidase and other mRNAs. Mol. Genet. Metab..

[33] Hartman T.R., Qian S., Bolinger C., Fernandez S., Schoenberg D.R., Boris-Lawrie K. (2006). RNA helicase A is necessary for translation of selected messenger RNAs. Nat. Struct. Mol. Biol..

[34] Wedeken L., Singh P., Klempnauer K.H. (2011). Tumor suppressor protein Pdcd4 inhibits translation of p53 mRNA. J. Biol. Chem..

[35] Su H., Xu T., Ganapathy S., Shadfan M., Long M., Huang T.H., Thompson I., Yuan Z.M. (2014). Elevated snoRNA biogenesis is essential in breast cancer. Oncogene.

[36] Fu L., Ma W., Benchimol S. (1999). A translation repressor element resides in the 3′ untranslated region of human p53 mRNA. Oncogene.

[37] Chen J., Kastan M.B. (2010). 5′-3′-UTR interactions regulate p53 mRNA translation and provide a target for modulating p53 induction after DNA damage. Genes Dev..

[38] Schumacher B., Hanazawa M., Lee M.H., Nayak S., Volkmann K., Hofmann R., Hengartner M., Schedl T., Gartner A. (2005). Translational repression of C. elegans p53 by GLD-1 regulates DNA damage-induced apoptosis. Cell.

[39] Koh D.C., Wong S.M., Liu D.X. (2003). Synergism of the 3′-untranslated region and an internal ribosome entry site differentially enhances the translation of a plant virus coat protein. J. Biol. Chem..

[40] Gasco M., Shami S., Crook T. (2002). The p53 pathway in breast cancer. Breast Cancer Res..

[41] MacGrogan D., Bookstein R. (1997). Tumour suppressor genes in prostate cancer. Semin. Cancer Biol..

[42] MacInnes A.W., Amsterdam A., Whittaker C.A., Hopkins N., Lees J.A. (2008). Loss of p53 synthesis in zebrafish tumors with ribosomal protein gene mutations. Proc. Natl. Acad. Sci. USA.

[43] Montanaro L., Trere D., Derenzini M. (2008). Nucleolus, ribosomes, and cancer. Am. J. Pathol..

[44] Yoon A., Peng G., Brandenburger Y., Zollo O., Xu W., Rego E., Ruggero D. (2006). Impaired control of IRES-mediated translation in X-linked dyskeratosis congenita. Science.

[45] Meng Z., Jackson N.L., Choi H., King P.H., Emanuel P.D., Blume S.W. (2008). Alterations in RNA-binding activities of IRES-regulatory proteins as a mechanism for physiological variability and pathological dysregulation of IGF-IR translational control in human breast tumor cells. J. Cell Physiol..

[46] Jeffers J.R., Parganas E., Lee Y., Yang C., Wang J., Brennan J., MacLean K.H., Han J., Chittenden T., Ihle J.N. (2003). Puma is an essential mediator of p53-dependent and -independent apoptotic pathways. Cancer Cell.

[47] Garrison S.P., Phillips D.C., Jeffers J.R., Chipuk J.E., Parsons M.J., Rehg J.E., Opferman J.T., Green D.R., Zambetti G.P. (2012). Genetically defining the mechanism of Puma- and Bim-induced apoptosis. Cell Death Differ..

[48] Kuilman T., Michaloglou C., Mooi W.J., Peeper D.S. (2010). The essence of senescence. Genes Dev..

[49] Lee C.G., Eki T., Okumura K., Nogami M., Soares Vda C., Murakami Y., Hanaoka F., Hurwitz J. (1999). The human RNA helicase A (*DDX9*) gene maps to the prostate cancer susceptibility locus at chromosome band 1q25 and its pseudogene (*DDX9P*) to 13q22, respectively. Somat. Cell Mol. Genet..

[50] Zhang S., Hemmerich P., Grosse F. (2007). Werner syndrome helicase (WRN), nuclear DNA helicase II (NDH II) and histone gammaH2AX are localized to the centrosome. Cell Biol. Int..

[51] Zhang S., Schlott B., Gorlach M., Grosse F. (2004). DNA-dependent protein kinase (DNA-PK) phosphorylates nuclear DNA helicase II/RNA helicase A and hnRNP proteins in an RNA-dependent manner. Nucleic Acids Res..

[52] Neplioueva V., Dobrikova E.Y., Mukherjee N., Keene J.D., Gromeier M. (2010). Tissue type-specific expression of the dsRNA-binding protein 76 and genome-wide elucidation of its target mRNAs. PLoS ONE.

[53] Van Maerken T., Rihani A., Van Goethem A., De Paepe A., Speleman F., Vandesompele J. (2013). Pharmacologic activation of wild-type p53 by nutlin therapy in childhood cancer. Cancer Lett..

[54] Brown C.J., Lain S., Verma C.S., Fersht A.R., Lane D.P. (2009). Awakening guardian angels: Drugging the p53 pathway. Nat. Rev. Cancer.

[55] Tovar C., Rosinski J., Filipovic Z., Higgins B., Kolinsky K., Hilton H., Zhao X., Vu B.T., Qing W., Packman K. (2006). Small-molecule MDM2 antagonists reveal aberrant p53 signaling in cancer: Implications for therapy. Proc. Natl. Acad. Sci. USA.

[56] Barone G., Tweddle D.A., Shohet J.M., Chesler L., Moreno L., Pearson A.D., Van Maerken T. (2014). MDM2-p53 interaction in paediatric solid tumours: Preclinical rationale, biomarkers and resistance. Curr. Drug Targets.

[57] Aziz M.H., Shen H., Maki C.G. (2011). Acquisition of p53 mutations in response to the non-genotoxic p53 activator Nutlin-3. Oncogene.

[58] Saiki A.Y., Caenepeel S., Cosgrove E., Su C., Boedigheimer M., Oliner J.D. (2015). Identifying the determinants of response to MDM2 inhibition. Oncotarget.

[59] Stegh A.H. (2012). Targeting the p53 signaling pathway in cancer therapy—The promises, challenges and perils. Expert Opin. Ther. Targets.

[60] De Benedetti A., Graff J.R. (2004). eIF-4E expression and its role in malignancies and metastases. Oncogene.

[61] Ghosh B., Benyumov A.O., Ghosh P., Jia Y., Avdulov S., Dahlberg P.S., Peterson M., Smith K., Polunovsky V.A., Bitterman P.B. (2009). Nontoxic chemical interdiction of the epithelial-to-mesenchymal transition by targeting cap-dependent translation. ACS Chem. Biol..

[62] Jia Y., Polunovsky V., Bitterman P.B., Wagner C.R. (2012). Cap-dependent translation initiation factor eIF4E: An emerging anticancer drug target. Med. Res. Rev..

[63] Li S., Jia Y., Jacobson B., McCauley J., Kratzke R., Bitterman P.B., Wagner C.R. (2013). Treatment of breast and lung cancer cells with a N-7 benzyl guanosine monophosphate tryptamine phosphoramidate pronucleotide (4Ei-1) results in chemosensitization to gemcitabine and induced eIF4E proteasomal degradation. Mol. Pharm..

[64] Moerke N.J., Aktas H., Chen H., Cantel S., Reibarkh M.Y., Fahmy A., Gross J.D., Degterev A., Yuan J., Chorev M. (2007). Small-molecule inhibition of the interaction between the translation initiation factors eIF4E and eIF4G. Cell.

[65] Chen L., Aktas B.H., Wang Y., He X., Sahoo R., Zhang N., Denoyelle S., Kabha E., Yang H., Freedman R.Y. (2012). Tumor suppression by small molecule inhibitors of translation initiation. Oncotarget.

[66] Yi T., Kabha E., Papadopoulos E., Wagner G. (2014). 4EGI-1 targets breast cancer stem cells by selective inhibition of translation that persists in CSC maintenance, proliferation and metastasis. Oncotarget.

[67] Papadopoulos E., Jenni S., Kabha E., Takrouri K.J., Yi T., Salvi N., Luna R.E., Gavathiotis E., Mahalingam P., Arthanari H. (2014). Structure of the eukaryotic translation initiation factor eIF4E in complex with 4EGI-1 reveals an allosteric mechanism for dissociating eIF4G. Proc. Natl. Acad. Sci. USA.

[68] Avdulov S., Li S., Michalek V., Burrichter D., Peterson M., Perlman D.M., Manivel J.C., Sonenberg N., Yee D., Bitterman P.B., Polunovsky V.A. (2004). Activation of translation complex eIF4F is essential for the genesis and maintenance of the malignant phenotype in human mammary epithelial cells. Cancer Cell.

